# Music emotion classification based on random swap algorithm

**DOI:** 10.1038/s41598-025-22336-0

**Published:** 2025-11-03

**Authors:** Abigail Wiafe, Sami Sieranoja, Pasi Fränti

**Affiliations:** School of Computing, University of Eastern Finland, P.O. Box 111, Joensuu, FI-80101 Finland

**Keywords:** Emotion recognition, Emotify+, Music, Clustering, Classification and taxonomy, Machine learning, Computer science

## Abstract

We performed an unsupervised cluster analysis of emotions in music. We considered ten distinct perceived emotions (happy, sad, amusing, annoying, anxious, relaxing, dreamy, energizing, joyful, and neutral) from four genres (rock, pop, classical, and electronic). We first clustered emotions based on their co-occurrence across songs, revealing that the emotion pairs of amusing/annoying, energizing/anxious, and relaxing/sad exhibited a strong correlation. We then clustered the songs into eight groups using the random swap clustering algorithm based on their emotional profiles (the distribution of emotion ratings for each song). The results revealed six clusters with one dominant emotion (relaxing, annoying, happy, amusing, anxious, and dreamy), one cluster with no emotion (neutral), and one cluster with an equal distribution of all emotions. Notably, some song clusters included both positive and negative emotions, thereby highlighting the presence of mixed emotional responses to music. The results are informative for future studies on emotions and emotional responses to music. These findings deepen our understanding of emotions evoked by music and provide a foundation for advancing research in music emotion recognition (MER). The same approach can also be applied to other types of media, such as poetry, painting, film music, and social media conversations.

**Supplementary Information:**

The online version contains supplementary material available at 10.1038/s41598-025-22336-0.

##  Introduction

Music affects the physiological processes in humans by stimulating adrenaline production, which can lead to excitement and energy. It can increase performance on cognitive tasks^1^ and create a sense of connection with loved ones^2^. Musical features such as tempo, rhythm, loudness, pitch, familiarity, listening repetition, and listeners’ affective experiences^3^ arouse profound emotions in listeners to regulate their mood states. Morley^4^ stated that although music is pervasive across all cultures, it is not only an auditory experience but also reflects intentional and momentary bodily action.

Emotions in music are often interrelated and can reflect complex relationships, owing to factors such as individual experiences, culture, environment, and contextual influences. Music may trigger mixed emotions in listeners, with both positive and negative feelings occurring simultaneously^5,6^. Berrios et al.^7^ provided empirical evidence suggesting that these mixed emotions are not merely experimental artefacts, but rather robust, measurable and genuine psychological experience. Understanding this emotional complexity is critical as it reflects the broader concept of emotional interconnectedness, which is explained through psychological theories and physiological responses. Moreover, there is a distinction between *perceived* emotions (the emotions a listener interprets from music) and *induced* (felt) emotions, which the listener experiences. These two can differ^6^. Although music can trigger either positive (e.g., happy) or negative (e.g., sad) emotions, understanding these emotional responses deepens the insight into how listeners relate to the different emotions experienced in music.

*Music emotion recognition (*MER) is a subfield of music information retrieval (MIR) that aims to predict emotions expressed in music using signal processing and machine learning techniques. It has attracted increasing attention in both academia and industry, and is used in psychotherapy, video games, recommendation systems, algorithmic music composition, automatic playlist generation, and music visualization.

Studies on emotion recognition in music have primarily been conducted using *categorical* and *dimensional* models. The categorical model uses discrete labels or adjectives such as happiness, fear, and anger to classify emotions^8^, which listeners find intuitive because they are familiar with concepts such as joyful and sad. In a dimensional model, emotions are represented using a dimensional space such as a 2D model^9,10^ or a 3D model^11–13^. Russell^14^ introduced a two-dimensional model, in which the dimensions were represented by arousal (exciting/calming) and valence (positive/negative). The three-dimensional model proposed by Mehrabian^15^ maps various continuous dimensions such as pleasure-arousal-dominance (PAD). Huo and Ge^16^ and Klonsky et al.^17^explored various multidimensional methods, but the number of dimensions that best fit the analysis of human emotional experience is not clear. In addition to basic emotions, domain-specific frameworks such as the Geneva Emotional Music Scale were developed to capture a more extensive range of emotions elicited by music^5^.

*Dynamic* and *static* methods have been used to classify and recognize emotions in music. The dynamic method assesses the changes in emotions over the course of a composition, for instance, every 0.5–1 s^18^, whereas the static method detects emotions in a relatively long section of the music of 15–60 secs^10^.

In this study, we used the static method to detect perceived emotions after listening to 60-second song excerpts. Emotional data were collected for emotion classification using the categorical emotion model, in which listeners rated the intensity of each emotion on a rating scale. We studied the Emotify dataset, consisting of 400 song excerpts of 60-second clips in four musical genres (rock, pop, classical, and electronic), using a web platform to investigate the impact of music on emotions. We created an extension of the Emotify dataset, called Emotify+, which consists of participants’ emotion annotations and their intensities.

Annotations were collected using a web-based music player called the EF Music tool. The tracks were ranked according to their genres, using their original order. We considered two possibilities for order: (1) randomization and (2) sorting by genre. The second choice was chosen because it makes the music collection easier to navigate, and users can better find the songs they prefer to listen to. This navigation method is closer to how users navigate in music listening applications such as Spotify. This was considered more motivating. The drawback is that earlier songs are annotated more often than later songs. This may have caused some hidden bias in the results, but it mostly affected the number of times songs were annotated. A song raising happy feelings would most likely do so, regardless of whether it was annotated five or 20 times.

One key aspect of designing the data tool is the selection of emotions from which users can choose. According to classical psychological theories of emotions in music^19^ and arguments posited by Chiang et al.^20^, there are no standardized rules for selecting emotional labels for use. We aimed to maintain a nuanced yet manageable set of options. Therefore, selected ten emotion annotations aligned with prior research, where the emotions varied from 4^21^ to 13^22^. Ten distinct emotion labels (*happy*,* sad*,* amusing*,* annoying*,* anxious*,* relaxing*,* dreamy*,* energizing*,* joyful*,* and neutral*) were selected based on previous studies on music emotions^22,23^. A few less–common emotions from Warrenburg’s taxonomy, such as tension, tender, and nostalgic, were excluded to maintain a focused and non-redundant set. These emotions were considered to be too nuanced or overlapped with the selected categories in the context of our study. The aim was to ensure that the emotions were labelled clearly and consistently.

Notably, we included joyful as a distinct label from happy to capture intense positive affect. Despite the overlap between these two emotional states, we opted to retain both to emphasize a subtle yet significant difference in emotional intensity, which is consistent with prior studies that distinguish between moderate- and high-arousal positive emotions, ensuring both sensitivity and consistency in our classification of emotions. The selected emotions were rated using a 5-point Likert scale, where listeners chose from a list of star rating options (1–5) to indicate the intensity of each emotion perceived in a song.

Importantly, this study investigates whether clustering analysis can uncover meaningful patterns in emotions evoked by music. Our research question is: *Do songs form clusters that are characterized by distinct emotional profiles?* We hypothesis that songs would cluster according to emotional valence, forming distinct groups of positive or negative emotions. For instance, songs frequently perceived as happy or joyful (positive emotions) would cluster separately from those predominantly perceived as sad or anxious (negative emotions).

To explore the co-occurring emotions, identify emotion patterns, and examine relationships among the emotions perceived, we applied the Random Swap clustering algorithm^24^. This algorithm is a variant of K-means known for improved clustering accuracy. Here, we leverage it to cluster music emotion data for the first time, which is a novel methodological approach within the MER landscape. By applying this technique, we aim to uncover new patterns in the emotional profiles of songs. The study also highlights the co-occurrence of positive and negative emotions within the same song cluster, challenging traditional dichotomies that strictly categorize emotions as positive or negative. Additionally, this study extends the Emotify + dataset to provide a richer emotional spectrum using emotion profiles to categorize music.

In this study, we constructed a co-occurrence matrix from the emotions annotated in the Emotify + database by counting the number of times emotions were perceived by listeners in the same song. We then applied clustering analysis in two different ways. First, we clustered the emotions and compared the results obtained with other datasets (both music and language) from the literature. Second, we constructed emotion profiles by counting the number of times that each emotion was perceived in a song. The songs were then clustered based on their normalized emotion profiles using the random swap clustering algorithm^24^. To the best of our knowledge, this is the first study that applies clustering to music pieces based on their emotional profiles.

The rest of the paper is organized as follows: Sect. “Related work on clustering and emotion analysis” presents a review of related work on the clustering of emotions and songs. The Emotify + dataset is discussed in Sect. “Dataset”. Section “Methodology” elaborates on the methodology, detailing the annotation procedure, the participants involved, and the clustering algorithm utilized. The results, discussion, and conclusions are outlined in Sects. “Results”, “Discussion”, and “Conclusion”, respectively.

##  Related work on clustering and emotion analysis

A cluster is a group of objects that are similar to each other, and objects in different clusters are expected to be less similar to each other. Cluster analysis usually consists of first separating the data objects into groups using a clustering algorithm and then analyzing the contents of these groups. Clustering can be utilized to summarize large amounts of data that are difficult to manually analyze.

Clustering has been used in various fields such as healthcare^25,26^, social networks^27^, classification and detection of emotions^28^, predicting the genre of music using the features of wave signals of music^29^, grouping and analyzing musical features from a set of preludes by Bach and Chopin^30^, and melodies in audio music^31^. Bakhshizadeh et al.^32^ proposed a framework to extract users’ mood by clustering their music track history. The clustering led to the generation of personalized music playlists based on the audio features of the music track.

Wang et al.^33^ proposed a multi-label music recognition system that uses a hierarchical Dirichlet process mixture model (HDPMM) to capture and analyze various emotional states in music. Using a discriminant factor and weighting coefficient, the system demonstrated an improved performance in recognizing and distinguishing emotions in music. Lupea et al.^34^ utilized hierarchical clustering to identify emotional patterns in Romanian poetry and ancient texts. This study discovered the emotional patterns associated with thematic content. Zhang and Sun^35^ identified emotion recognition in web music and classified music into specific emotional labels using reverse gene expression programming. Although there was an improvement in the classification accuracy and processing time, an appropriate feature selection method was required.

Toivonen et al.^36^ investigated the clustering of Finnish words that describe emotional experience. Their study suggested that the application of hierarchical clustering aids in assessing extensive emotional concepts or combinations of emotions. It also suggested that the study was the first to use clustering to describe the relationships between Finnish emotional concepts identified at different levels.

Wong et al.^37^ used hierarchical clustering to analyze the structure and dimensions of the Cantonese emotion lexicon. This study identified clusters related to emotional words based on their emotional features. It also explored different cluster solutions using different distance metrics and linkage functions to provide in-depth knowledge of how Cantonese emotional words are grouped based on their emotional features.

In summary, existing literature has reported the application of hierarchical agglomerative clustering in the classification of emotions. In this study, we applied the same approach to the Emotify + dataset to determine whether our data could confirm previous findings.

##  Dataset

The study utilizes the Emotify + dataset^38^, which is an extension of the Emotify music dataset^39^ consisting of 400 songs (44100 Hz, 128 kbps, one minute each) from four different genres: classical, rock, pop, and electronic music (100 tracks per genre). Each song excerpt came with a predefined genre label provided by the dataset creators.

Participants were instructed to select the emotion(s) that they most strongly perceived while listening to each song excerpt, selecting from a predefined set of emotion labels the emotion that best reflected their subjective experiences. Although joyful was included as a label distinct from happy to capture intense positive emotions, there was an overlap between these two emotional states. Both labels were retained to reflect a subtle nuance in emotional intensity and maintain alignment with prior studies that also distinguished between these closely related but non-identical affective states. Although the Emotify music dataset consists of 400 songs, a detailed analysis revealed that the dataset had 390 unique songs with 10 repeated songs. The pop, classical, and rock genres had two, three, and five songs, respectively, repeated twice. Participants provided 3031 emotion annotations.

## Methodology

### Participants

A total of 181 volunteers (140 males and 41 females) participated, each listening to multiple songs and noting the emotions they perceived. This study was approved by the review committee of the University of Ghana. The participants were informed about the research purpose and consented to the use of their anonymized emotion ratings. No personal data was stored. Basic demographics such as gender and nationality were recorded, but per GDPR, these were not considered personal identifiers.

### Annotation procedure

We used the Emotify music dataset^39^, which consists of 400 songs (44100 Hz, 128 kbps, one minute each) from four different genres: classical, rock, pop, and electronic music. The songs were in English, and the files were in mp3 format. The EF Music tool, developed by the machine learning group at the University of Eastern Finland, was used to collect the emotional data. Participation was voluntary without any reward. Participants were invited to listen to the selected songs and annotate their emotions perceived and not induced while using our customized EF Music tool (a web application). Participants had the option to perform a listening task at their convenience using their device (phone or computer). Given the selected list of emotions, a listener can freely choose an emotion that he/she perceive and rate its intensity. The instructions to use the music player were as follows.

- The participants are given a link to the webpage, instructed to register as a user, and then listen to any music he/she wishes to listen to. It was not required to listen to all 400 songs, but as many as the participants felt like doing.
- After listening, the participant can select any emotion from the list and rate its intensity on a Likert scale from 1 to 5. There were no limitations to the number of emotions that could be annotated to the same song.
- Participants can skip listening and go to another song or genre at any time.
- If no emotion is perceived, the participant can either skip the annotation of that song or select ‘Neutral’ from the list of emotions.

**Fig. 1:**
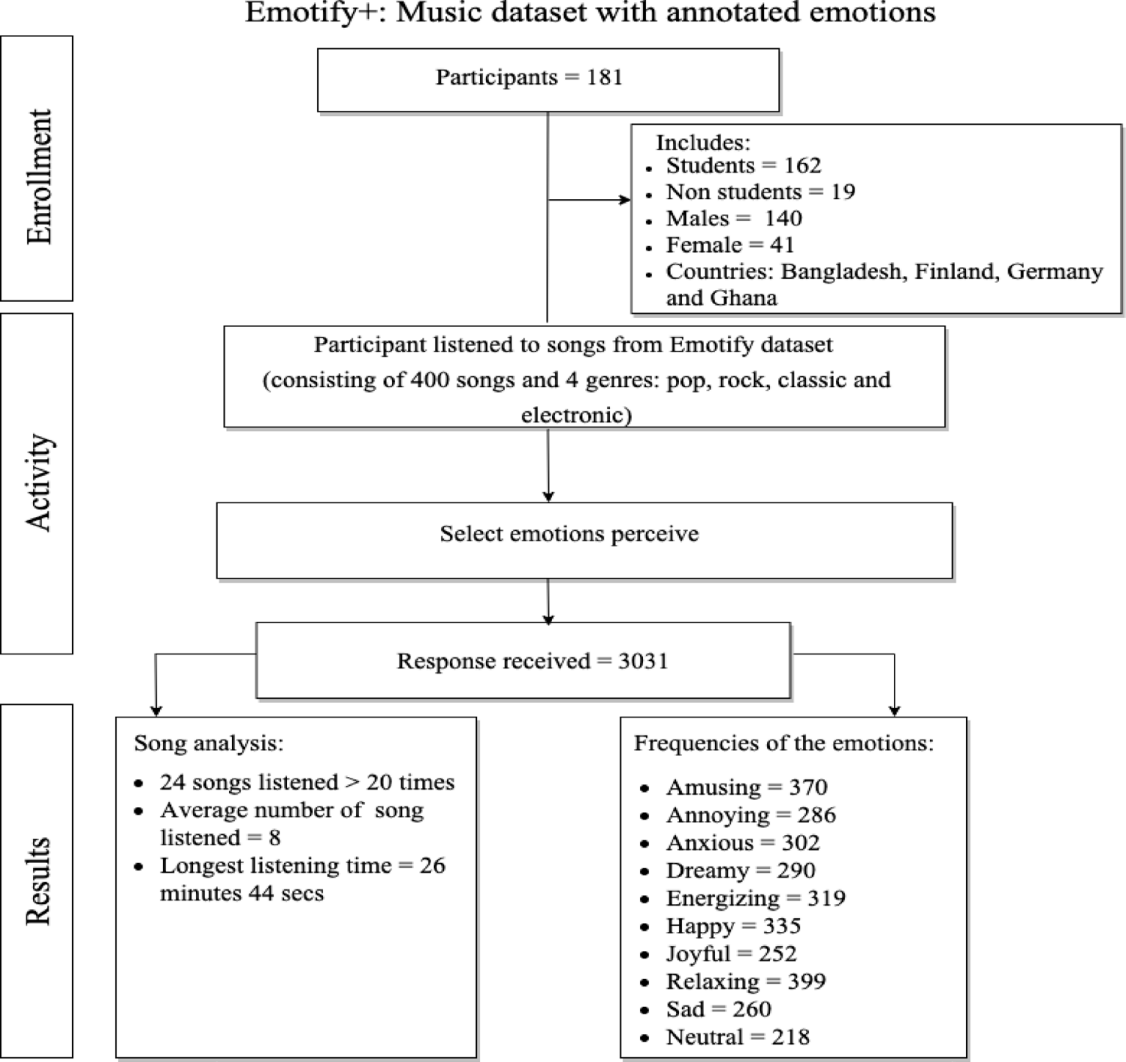
Data gathered.

To ensure data reliability, multiple quality control measures were implemented during data collection. The participants were required to register with a valid email address to prevent duplicate accounts. Listening durations were automatically recorded for each annotation, enabling the monitoring of participant engagement and the filtration of the listening time(s) where participants selected emotions within an unrealistically short time (e.g., less than 5 s) unless verified by the system. In addition, annotations that were not in line with reasonable listening behaviour (such as multiple annotations within a short time) were flagged and checked manually, but no instances of fraudulent behaviour were discovered. Figure 1 provides an overview of the activities involved and the data gathered during the creation of the Emotify + dataset.

### Analysis method

To analyze the emotional structure of the music data, we employed the random swap clustering algorithm^24^, an enhanced variant of the K-means algorithm. Random swap was chosen due to demonstrated stability, robustness and superior clustering accuracy in complex datasets, particularly those with overlapping or imbalanced clusters. Unlike k-means, which is highly sensitive to initial centroid position and often coverages to local minima, the random swap employs a stochastic exploration mechanism to avoid local minima and explores a wider solution space. The algorithm iteratively replaces existing cluster centres with randomly selected data points (swaps) and then applies K-means refinement after each swap. A swap is accepted only if it improves the clustering quality, typically measured by the within-cluster sum of squares (WCSS).

##  Results

### Clustering emotion labels

The clustering algorithms used were not new. However, the strength of using well-established and tested methods is that there is more confidence in the results than when using a completely new algorithm that has not been extensively tested on other datasets and cases. The Silhouette coefficient (SC) values are affected by both the quality of clustering and characteristics of the data. For example, if the similarities between data objects are completely random, then the SC value would be zero regardless of the chosen methodology. As the performance of the chosen clustering algorithms has been well tested in many previous benchmarks, we believe that the characteristics of the data led to relatively low values of SC.

To optimize the clustering parameters, we used two algorithms:

i. Hierarchical agglomerative clustering algorithm with average linkage criteria.
ii. Random swap algorithm (improved version of K-means).

The first is completely deterministic; therefore, there are no differences between different runs. The second utilizes randomness, but when running it extensively, for 5000 swaps, the results converged to the same solution across different runs. The algorithm does not require tuning the parameters.

**Fig. 2:**
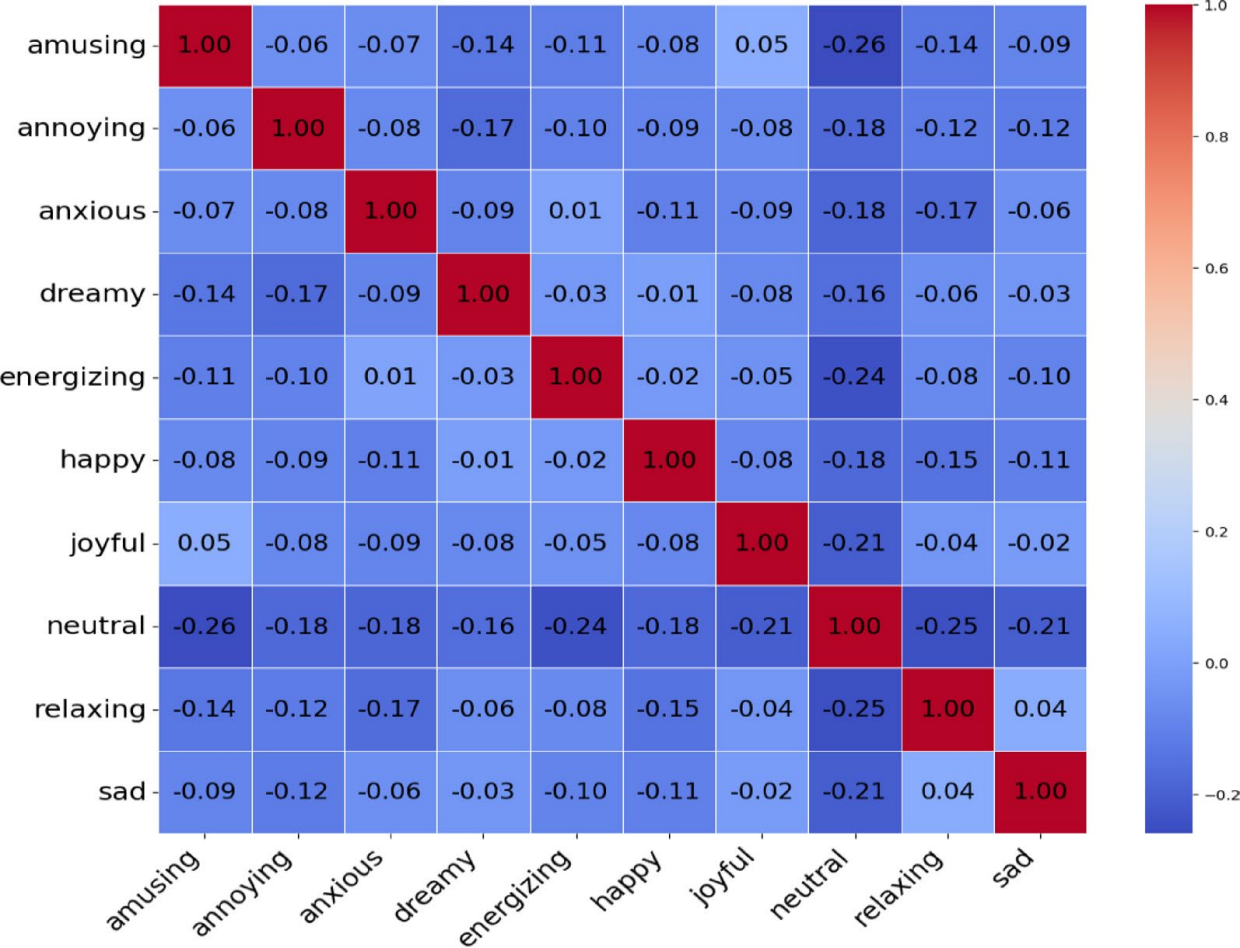
Emotion similarity matrix based on Pearson correlations. Warmer colours indicate stronger co-occurrence in listener ratings.

To cluster the emotions, we calculated a similarity matrix between the emotions, as shown in Fig. 2, and used it as input to the algorithm. We first created the emotion profiles of the songs as follows: the number of times a song was rated with a certain emotion was transformed into percentage values that sum up to 100% for each song. We then calculated the Pearson correlation coefficient between each pair of emotions in the song profiles to construct a similarity matrix (see Fig. 2). Because each song’s emotion profile is compositional (all values sum up to 100%), many of the correlations between emotion labels were negative or near zero. The range of correlations in the matrix was − 0.26 to 0.05. Values larger than − 0.10 are considered to signify a close relation between the emotions. The correlation values (similarities) are then converted to distance values for clustering using distance = 1 – correlation.

**Fig. 3:**
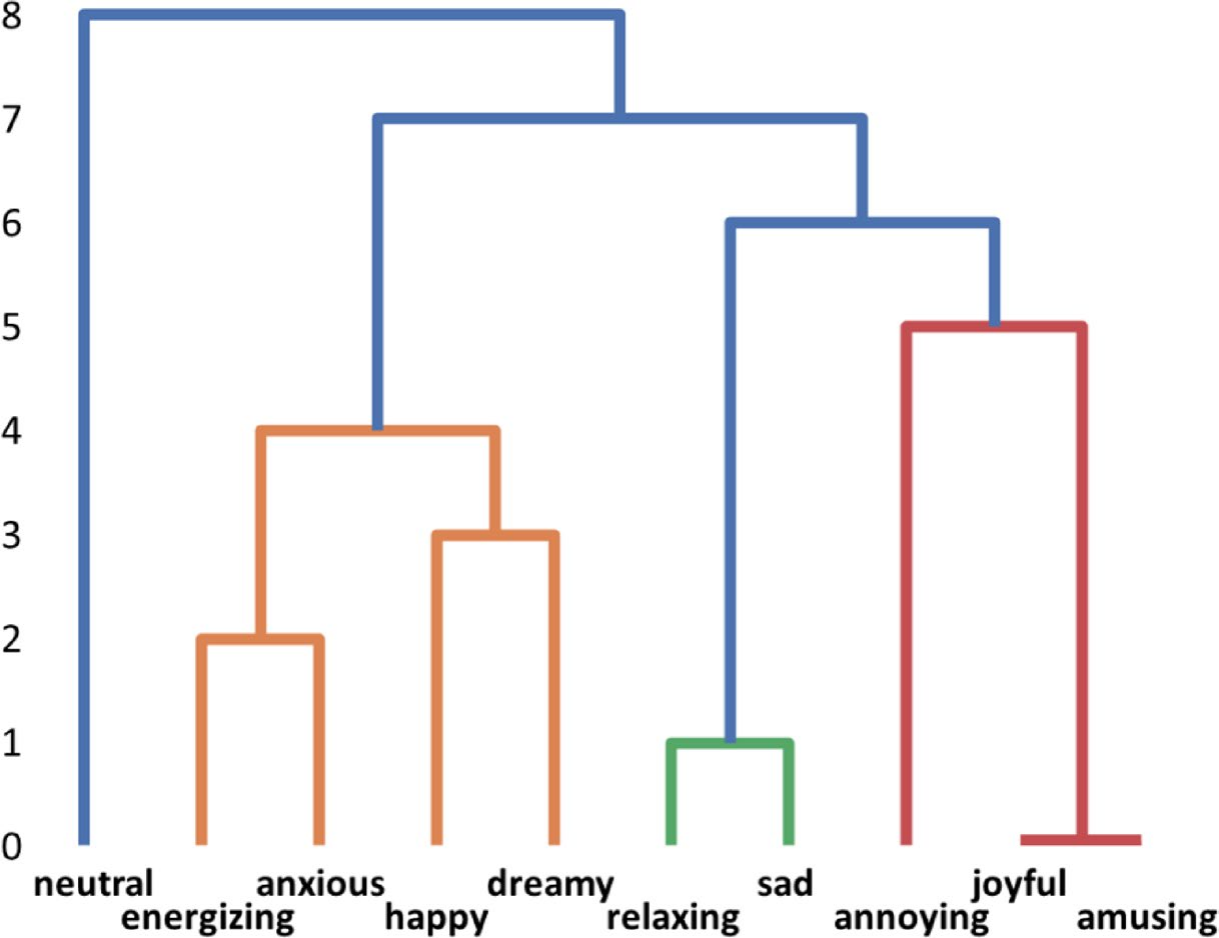
Dendrogram of emotions. The height of the horizontal lines determines the order of the merges. The lowest are merged first.

We used a hierarchical agglomerative clustering algorithm with average linkage criteria. Compared to other clustering methods, such as k-means clustering, it also provides a hierarchy of clusters that can be visualized as a tree-shaped dendrogram (Fig. 3). The branches of the dendrogram indicate the closeness of the clusters to other clusters. Agglomerative clustering is implemented using the scikit-learn library in Python.

The clustering process begins with each data object (emotion) in its own cluster. In each step, the algorithm merges the pair of clusters that are most similar to each other according to a cost function. We used the average linkage criteria (Eq. 1), because it has been recommended for psychological data^40^. The merging process stops when all the objects merge into a single cluster.

The average linkage cost function is defined as follows:

1 $$\:cost\left(A,B\right)=\frac{1}{{n}_{A}\cdot\:{n}_{B}}\sum\limits_{i=1}^{{n}_{A}}\sum\limits_{j=i}^{{n}_{B}}d({x}_{Ai},{x}_{Bj})$$

where $\:{n}_{A}$ is the size of cluster *A* and $\:d({x}_{Ai},{x}_{Bj})$ is the distance between the *ith* object in cluster A and the *jth* object in cluster B.

The clustering results are presented in Fig. 3 as a dendrogram. The similarity matrix (Fig. 2) provided by the clustering algorithm was also useful for analysis. Both display connections between emotions; however, the most important connections are easier to observe in clustering results.

In the dendrogram, more similar emotions were merged earlier. The vertical lines in the dendrogram illustrate the grouping of the clusters, and the horizontal bars indicate the points at which the two clusters merge. A dendrogram can be used to divide a dataset into a specified number of separate clusters by cutting it at a certain level. A suitable number of clusters can sometimes be determined by optimizing certain measures such as the Silhouette coefficient^41^. We calculated the Silhouette coefficient (SC) for all clusters between two and nine. The highest SC value (0.098) was observed for two clusters. However, such a small number of clusters is impractical for our data analysis. Given the small size of the dataset, manual examination of the dendrogram was more effective. By setting a cut-off at the sixth stage, we identified four clusters that provided a suitable level of precision for summarizing the data.

*Amusing* and *joyful* are the emotions that correlate most strongly (0.05) and, therefore, merged first in the clustering process. *Annoying* was most strongly correlated with amusing (−0.06), which shows that some songs that include humorous content cause irritation to some listeners and amusement to others. A similar combination of close relationships and positive/negative emotion polarity was also observed in the emotion pairs *relaxing/sad* (0.04) and *energizing/anxious* (0.01). Neutral had the most negative correlation with the other emotions and was the last to merge.

This makes sense because *neutral* is not an emotion but suggests a lack of conveyed emotions. This is likely to be chosen by listeners when no other emotions are suitable. In summary, the findings suggest that the emotion space in the music dataset is not split into purely positive and negative groups but rather includes a cluster of mixed-valence emotions.

### Clustering songs based on emotion profile

**Fig. 4:**
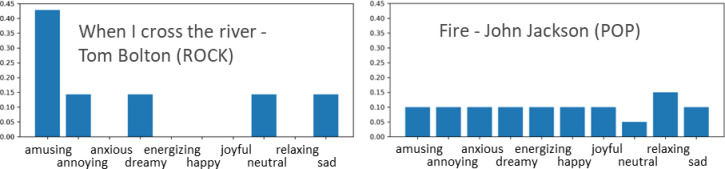
Example of emotion profiles for two songs.

**Fig. 5:**
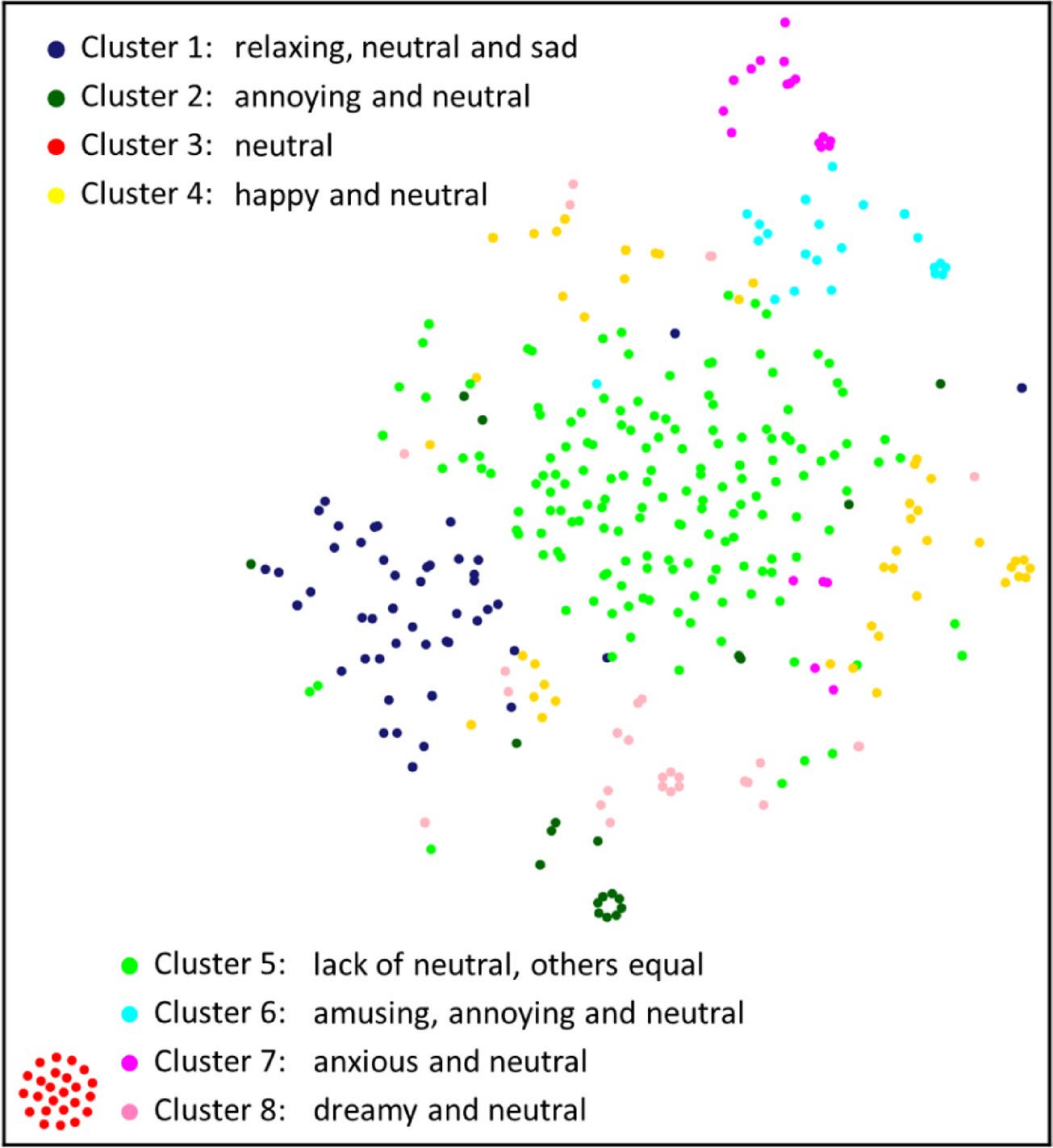
T-SNE projections of clustering results. The features are emotion profiles. The most prominent emotions are listed for each cluster.

In this section, we present the results of song clustering based on their emotional profiles. An example of the emotional profiles of two songs is shown in Fig. 4. The results are presented in Table 1; Fig. 4, respectively. The profile for a song was calculated by counting the number of ratings for each emotion and dividing them by the total number of ratings so that the values of the resulting vector sum up to 1.0. This profile can also be considered as an emotion probability distribution for the song. To visualize the dataset, the emotion profiles were converted into a 2D space using t-SNE (Fig. 5).

We clustered emotion profiles using the random swap algorithm, which was shown to provide significantly more accurate clustering results than standard k-means. We used the sum of squared errors (SSE) cost function and Euclidean distance. The number of swaps were set to 5000. The silhouette coefficient was used to determine the suitable number of clusters. The highest SC value in the range of k = 2–29 appeared in the case for the two clusters (0.34). However, this level of clustering did not provide sufficient detail regarding the data. Therefore, we selected k = 8, which was more convenient for analysis, and had the highest value (0.27) in the range of k = 2–29.

**Table 1:** Summary of clusters. A representative song is the song with an emotion profile that is closest to the average profile of the cluster. The song can be heard by following the link.

Cluster	Size	Prominent emotions	Representative song
1	56	relaxing, neutral, sad	Give me love - Norine Braun (POP) http://cs.uef.fi/ml/musicemotions/pop/59.mp3
2	22	annoying, neutral	Solitude (Khlas 6–8) - Solace (ELECTRONIC) http://cs.uef.fi/ml/musicemotions/electronic/61.mp3
3	24	Neutral	Till my cup Runs Over - Four Stones (POP) http://cs.uef.fi/ml/musicemotions/pop/23.mp3
4	50	happy, neutral	Zabudni na to - Strojovna 07 (ELECTRONIC) http://cs.uef.fi/ml/musicemotions/electronic/72.mp3
5	162	lack of neutral, other emotions mostly equal	Fire - John Jackson (POP) http://cs.uef.fi/ml/musicemotions/pop/24.mp3
6	27	amusing, annoying, neutral	When I cross the river - Tom Bolton (ROCK) http://cs.uef.fi/ml/musicemotions/rock/4.mp3
7	24	anxious, neutral	Insomnia - Processor (ELECTRONIC) http://cs.uef.fi/ml/musicemotions/electronic/56.mp3
8	34	dreamy, neutral	Introduction - Kourosh Dini (ELECTRONIC) http://cs.uef.fi/ml/musicemotions/electronic/17.mp3

**Fig. 6:**
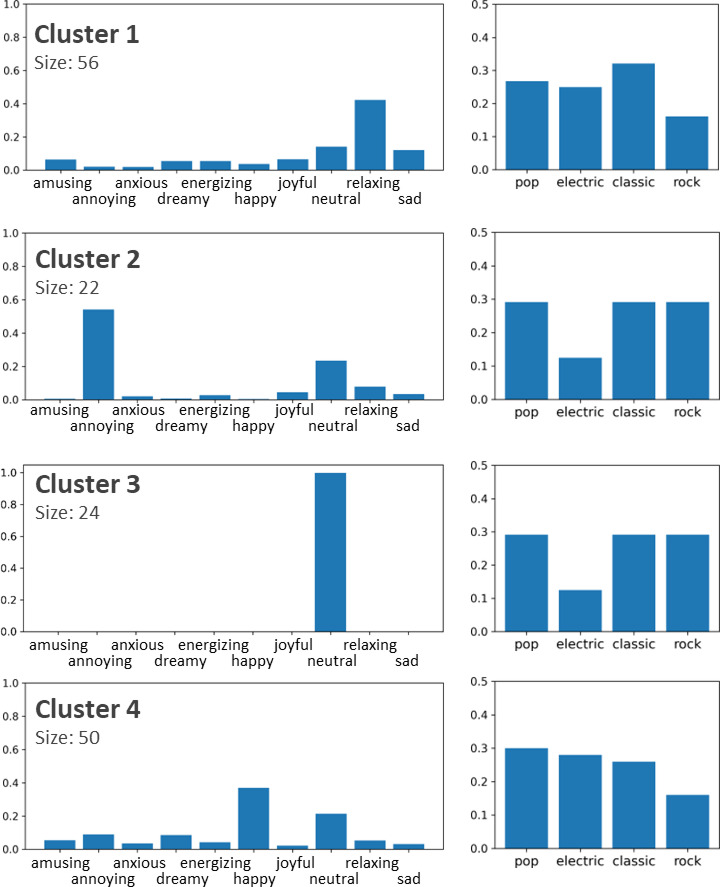
The first four of the eight clusters.

**Fig. 7:**
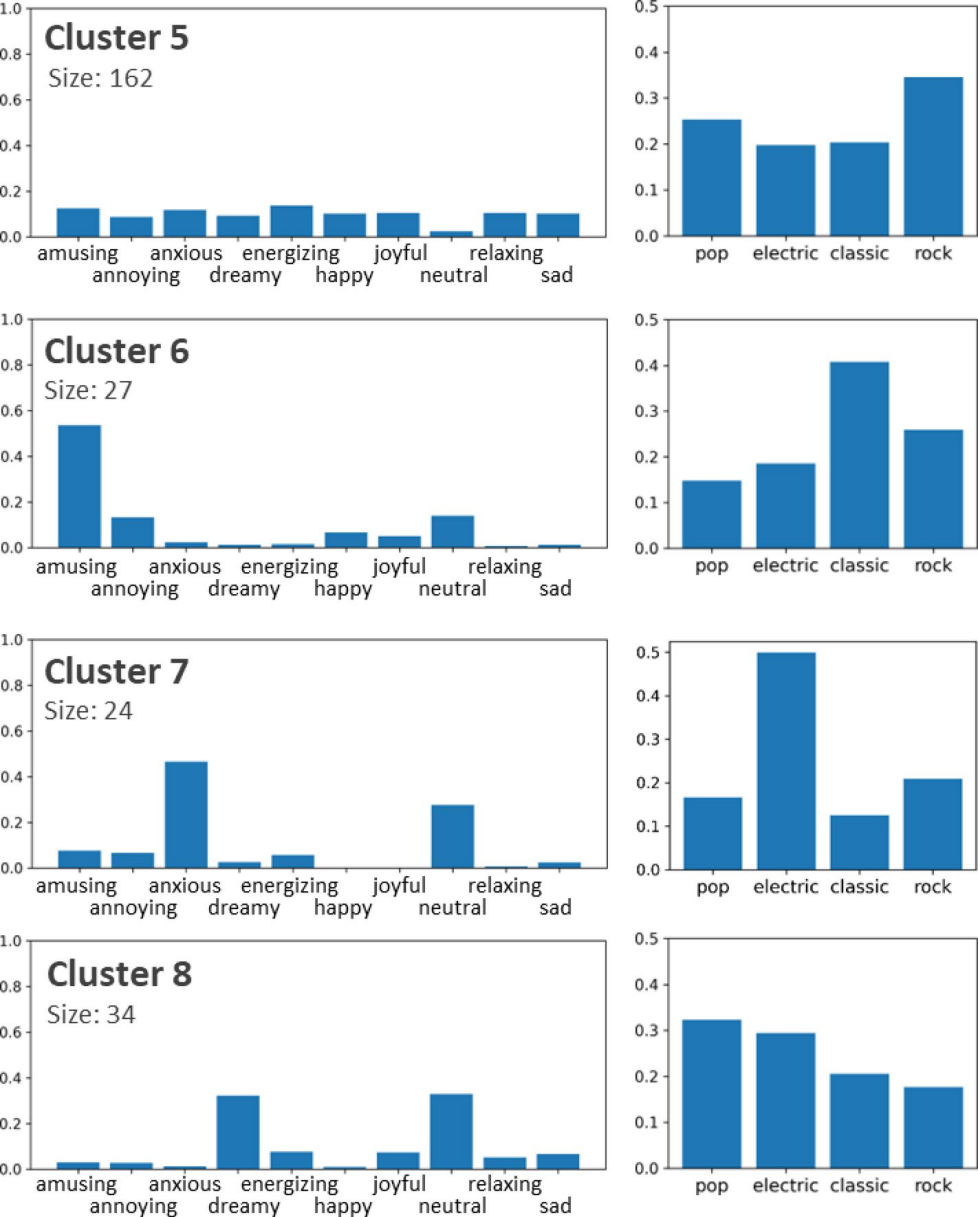
The last four of the eight clusters.

Figures 6 and 7 shows the eight clusters. Most of the clusters were formed around one or two key emotions. For example, cluster two includes songs that were mostly considered annoying or neutral. For Cluster 6, amusing was the most prominent emotion. This type of pattern can be observed in Clusters 1, 2, 4, 6, 7, and 8. A common property of these clusters is that neutral is always the second or third most prominent emotion. Therefore, for songs in these clusters, the user either recognized 1–2 key emotions or did not recognize any emotions.

In contrast to the previous case, Cluster 5 had neutral as the least prominent emotion, and other emotions had roughly equal prevalence. Cluster 5 was also the largest cluster, with 162 songs, and contained 40% of the data. Therefore, the entire dataset contains two distinct groups: songs with one or two clear key emotions (clusters 1, 2, 4, 6–8) and songs that evoke a larger range of emotions. In addition, the data contain one outlier cluster for songs that have neutral as the only emotion, that is, the user cannot recognize any emotion when listening to these songs.

In the emotion profiles of clusters, the most prominent emotions usually have logical connections. For example, in Cluster 6, the most prominent emotions were amusing and annoying. This means that songs that amuse some listeners are experienced as annoying by others. The same observation can be made without clustering analysis directly from the correlation matrix shown in Fig. 2. However, not all observations from cluster analysis correspond to the data in the correlation matrix. For example, Fig. 2 shows that neutral emotion had the most negative correlation with other emotions. Cluster analysis showed that this was indeed the case for some songs, but only for those (46% of the data) in Clusters 3 and 5. For other clusters, neutral was typically the second most prominent emotion and, therefore, in those cases, had a high correlation with many other emotions.

### Genre-Based emotion profile

To identify the emotion profiles for each genre, we counted the emotion ratings for each genre and divided them by the total number of ratings for that genre. Each genre exhibits a distinct emotional profile. For instance, classical music was most frequently described as relaxing (17%) and amusing (16%) than the other genres. This finding implies that classical music can incite calmness and pleasure. Electronic music was rated as the most anxious (13%) and annoying (11%) among the genres. They were also perceived as the most neutral (12%), least joyful (8%), and least sad (6%). These findings indicate that electronic music can trigger mixed emotional responses with a preference for neutrality and annoyance instead of strong positive or negative emotions. Pop music was considered the happiest (12%) and had the smallest share of ratings for annoying (8%) and anxious compared to classical music (8%). In addition, it was the most energizing genre (12%).

With the emotion profile for each genre, positive emotions (joyful, happy, amusing, energizing, dreamy, relaxing) and negative emotions (sad, annoying, anxious), Classical and Pop music had the most positive emotions (11.2%, 11.3%), while Electronics and Rock had the least (9.8%, 10.8%). Electronic and Rock also had the highest mean values for negative emotions (10.4% and 9.8%, respectively), whereas Classical and Pop had the lowest (8.5% and 8.7%, respectively). Listeners experienced positive emotions more often than negative emotions when listening to song excerpts (64% vs. 28%). Similar findings were reported by Juslin et al.^42^, who observed that 84% of the emotions experienced during everyday music listening episodes were positive.

### Statistical analysis of emotion rating and Inter-Rater agreement

We performed a correlation analysis (using JASP software) to reveal a systematic relationship between the emotion categories. Neutral emotions negatively correlated with most other emotions. For instance, neutral showed correlation coefficients of (−0.26) with amusing, (−0.24) with energizing and (−0.25) with relaxing (all *p* < 0.001). This suggests that the more a listener experiences a neutral response to a song, the less likely they are to report feeling amusing, energized, or relaxed. Annoying and anxious were weakly related to other emotions; annoying had a weak negative correlation with energizing (−0.10, *p* < 0.05) and dreamy (−0.17, *p* < 0.001). Anxious was moderately negatively correlated with relaxing (−0.17, *p* < 0.001) and neutral (−0.18, *p* < 0.001). Relaxing did not strongly correlate with most others; it showed a small negative correlation with happy (−0.15, *p* < 0.01), amusing (−0.14, *p* < 0.01), and annoying (−0.12, *p* < 0.05), suggesting that feeling relaxed tends to diminish feelings of happiness and amusement slightly. Dreamy had a small negative correlation with amusing (−0.14, *p* < 0.01) and energizing (−0.03, not significant). This indicates a slight tendency for dreamy to be linked to reduced amusement.

We also assessed the reliability and diversity of the emotion annotations. The inter-rater reliability (Krippendorff’s alpha) for emotion labels was low (approximately 0.16), which reflects substantial variability in how different listeners categorize the same song. Furthermore, the variability in the emotional response was measured by computing the entropy of each song’s emotion label distribution. The distribution of emotional entropy was bimodal. A substantial proportion of songs had low entropy values (e.g., < 0.1 bits), indicating that the majority of listeners assigned the same emotion. In contrast, another substantial group exhibited near-maximal entropy (> 0.9), suggesting that emotional ratings were evenly distributed across multiple emotion categories. Based on this pattern, it is suggested that there are two predominant types of songs in the dataset: those that are emotionally clear and easily interpreted and those that are emotionally complex or ambiguous, which elicit different reactions from listeners. In addition, many songs evoke a consistent emotion across listeners or evoke no single dominant emotion.

Additionally, an exploratory factor analysis (EFA) was conducted to examine the latent structure of listeners’ emotional responses to music. One-hot encoding was applied to the ten emotional categories, generating a binary matrix of user-song interactions. Records without emotional input were excluded. Before extraction, data suitability was assessed using the Kaiser-Meyer-Olkin (KMO) measure of sampling adequacy, which yielded a value of 0.72, indicating that the data were appropriate for factor analysis (Kaiser, 1974). Factor extraction was performed using principal axis factoring with oblique rotation (Promax) to allow for correlation among factors. Based on the eigenvalues > 1 criterion and interpretability of the solution, three factors were retained (thus, the Kaiser-Meyer-Olkin (KMO) test value was 0.72, and Bartlett’s test of sphericity was significant at (χ²(45) = 298.67, *p* < 0.001), suggesting that the data was suitable for factor analysis). Three distinct factors were identified. Factor 1 was associated with a neutral-tone response (neutral). Factor 2 captured calm or smoothing emotional dimensions (e.g. relaxing, dreamy) and Factor 3 reflected positively activated emotions (e.g. happy, energizing and joyful). The emotion annoying did not load significantly on any of the three factors, indicating weak negative loading across all dimensions. This pattern suggests that annoying does not align with calming, activating, or neutral dimensions and is instead part of a negative affect cluster, alongside emotions like anxious and sad. Collectively, these three factors accounted for 62% of the total variance, indicating a robust underlying structure in emotional response data. Furthermore, factor analysis supports a three-dimensional emotional structure in music perception, with ‘annoying’ emerging as a distinct negative response, valuable for personalized affective systems.

Furthermore, to compare multiple emotions perceived across different songs, a repeated-measures Analysis of Variance (ANOVA) was conducted to examine the effect of listening to songs as independent variables, with expressed emotions (joyful, happy, amusing, energizing, dreamy, relaxing, sad, annoying, and anxious) as dependent variables.

Mauchly’s tests revealed that the assumption of sphericity had been violated (*p* < 0.05); therefore, Greenhouse-Geisser corrections were applied. The results demonstrated a significant effect of emotions expressed (F (9,3582) = 16.439, p = < 0.001, ω2 = 0.040).

A post-hoc test using the Bonferroni correction was performed to determine the emotions that significantly differ from each other. Multiple comparisons indicated a significant difference (***) between neutral and other emotions, suggesting that it differ significantly from other emotions. For instance, happy vs. neutral (*p* < 0.001) were significantly different. In contrast, emotion pairs such as joyful vs. anxious and sad vs. annoying showed no significant difference (*p* > 0.05). These results indicate that neutral emotion differed substantially from the other emotions (see Table A1 in the Appendix for more details).

This analysis complements the clustering results by revealing that certain emotions, such as neutral emotions, are generally more prevalent in music responses. This trend may explain the formation of specific clusters, such as a large cluster dominated by neutral responses. The per-emotion Krippendorff’s alpha values were also low, ranging from 0.14 to 0.18, indicating limited consensus among raters. The results suggest that some emotions were more reliably identified than others. This was evident across musical excerpts, where participants selected a diverse range of emotions rather than converging on a dominant choice.

##  Discussion

We selected the random swap algorithm to ensure more stable and robust clustering results, particularly considering the variability commonly associated with standard k-means clustering. Although traditional k-means can produce similar results when run with different initializations, a random swap offers more stable and improved accuracy by preventing poor local minima. The application enabled us to obtain consistent clustering across trials, increasing the reliability and interpretation of our findings.

While alternative clustering methods, such as model-based methods, could have been considered, our primary objective was to gain meaningful insights from emotion annotations rather than benchmarking the algorithm’s performance. Therefore, performing a comprehensive comparison of clustering methods was beyond the scope of this study. Nonetheless, the use of a random swap algorithm in this context is a novel methodological contribution within the MER domain and demonstrates efficiency in clustering emotionally annotated music data.

The clustering of emotions and songs provides several insights. Notably, the most prominent emotion in each cluster often exhibited meaningful relationships or contrasts. For instance, Cluster 6 was characterized by amusing and annoying emotions. This implies that songs entertaining to some listeners can be perceived as annoying by others. The same observation that a song excerpt can elicit opposite reactions can also be made directly from the emotion correlation matrix (Fig. 2), which demonstrates a negative correlation between amusing and annoying. However, not all patterns observed in the clustering were apparent in simple correlation analysis. For instance, (Fig. 2) showed that neutral emotion had a strong negative correlation with most other emotions. While clustering reflects this for some songs, particularly those in the neutral and mixed clusters (which together make up about 46% of the data), neutral occurs frequently as a secondary emotion in other clusters. In these instances, neutral correlates positively with many other emotions because it appears alongside them in the song profile. This highlights how clustering songs provides a complementary perspective: rather than capturing overall emotion correlations, it identifies specific groups of songs where certain emotion combinations are more prominent.

Additionally, we conducted an exploratory factor analysis (EFA) on the emotional data to identify the latent structure among the reported emotional responses. The analysis revealed three distinct factors. The first factor was associated with a neutral-tone response (neutral), whereas the second factor captured calm or smoothing emotional states, such as relaxing and dreamy. Positively activated emotions such as happy, energizing and joyful were captured by the third factor. Notably, happy and joyful exhibited strong loadings on the same factor, highlighting their semantic and perceptual similarities in the music context. Conversely, annoying was loaded separately, reflecting the distinct status of a felt reaction rather than a perceived musical emotion. These findings provide valuable insights into how listeners interpret emotional cues in music and how specific emotion terms or labels function within the MER dataset.

It is important to note that our dataset included a mix of perceived and felt emotional descriptors, which introduced some ambiguity. While participants were instructed to rate the emotion evoked by the music, that is, perceived emotion, some responses (e.g., annoyance) may reflect listeners’ own felt experiences. We explicitly state this as a limitation of the present study. Furthermore, we clarified that the list of emotions included both perceived and felt emotion descriptors, which might have introduced some inconsistencies in the responses. Although this mixture is not ideal, it is necessitated by the structure and constraints of the available datasets. It is acknowledged that the simplification was a methodological choice, and we remain transparent about its implications for data interpretation.

The dataset also presented imbalances related to genre distribution. Thus, because songs were listened to in a fixed genre sequence, genre contributed to an ordering effect; earlier genres received more total responses. Pop songs received higher ratings on average because of the fixed presentation or ordering of songs grouped by genre. This ordering effect led participants to encounter Pop and Rock genres (earlier genres received more responses). In contrast, Classical and Electronic genres had low ratings. This ordering effect introduces bias in the emotional annotation data across genres and is noted as a limitation.

With issues related to genre classification, genre labels in the dataset were sourced from the original metadata, and we agree that the genre labels were sometimes overly broad and may not always accurately represent the specific musical style of certain songs. However, because the study did not focus on genre analysis, we did not extensively reclassify genres.

Additionally, our engagement with music emotion literature has expanded, particularly in the interpretation of complex and unexpected emotional patterns. Drawing from existing research on mixed emotions in music, we recognize that musical pieces often evoke multiple or even conflicting emotional responses. Accordingly, Larsen and Stastny^43^ suggested that the conflicting emotional responses may arise from music that presents emotionally incongruent cues. For instance, listening to slow songs in major modes and fast songs in major modes, mixed emotions are expressed.

These conflicting findings support this view, revealing the co-occurrence of positive and negative emotions in song clusters and challenging the traditional view that musical emotions can be neatly divided into valenced categories. This evidence suggests that emotional responses to music are often nuanced, multifaceted, and subjective, which calls for more flexible models of emotional representation in music psychology.

##  Conclusion

In this study, we analyzed human emotional responses while listening to song excerpts. Our analysis employs two distinct cluster analysis techniques. Initially, we grouped emotions by leveraging the emotion ratings assigned to songs. Subsequently, we applied clustering to the songs based on their emotional profiles.

The cluster analysis yielded unexpected results. Contrary to our expectations, the emotions were not segregated into distinct valence-based groups. Instead, both positive and negative emotions frequently co-occur within the same cluster. This outcome stands in contrast to previous studies where emotions were found to cluster mainly by valence (for instance, Susino and Schubert^44^ reported that emotions grouped into purely positive or negative categories). Our data shows that the same musical piece can evoke a positive emotion in some listeners and negative emotions in others. For example, a slow-tempo song can evoke feelings of sadness in some, but relaxation in others, highlighting the subjective nature of emotions evoked by music. Our findings indicate that the primary distinction between songs lies in whether they evoke a clear, consensual emotion or a diffuse mixture of emotions among listeners. The presence of a large mixed cluster in our results suggests that many song excerpts do not fit perfectly into single-emotion categories, emphasizing the importance of recognizing mixed or complex emotional experiences in music psychology.

Our clustering-based approach, which applies a well-established algorithm in a new application domain, can easily be applied to other forms of media to investigate emotion induction. Similar methods could be utilized to group poems and visual works, film soundtrack excerpts, or social media posts based on emotional responses. Finally, the Emotify + dataset and the identified emotion-based clusters offer valuable directions for future work, particularly in exploring how these emotional groupings correlate with the underlying acoustic and musical features. Such investigations have the potential to enhance our understanding of the relationship between musical structure and emotional perception. Future research will examine whether specific audio features, such as mode, tempo, dynamic timbre, or other musical descriptors, can accurately predict whether a song belongs to an emotional cluster. This approach could help bridge the gap between the psychological clustering of emotional responses and computational analysis of musical structure, thereby advancing research in music emotion recognition and recommendation systems.

## Supplementary Information

Below is the link to the electronic supplementary material.

Supplementary Material 1

Supplementary Material 2

## Data Availability

The datasets generated and/or analyzed during the current study are available in the [Mendeley Data] repository [https://data.mendeley.com/datasets/wbk8zr9bd2/1].
