# Supplementary material for: Music emotion classification based on random swap algorithm
Source: Sci Rep. 2025 Nov 3;15:38337. doi: 10.1038/s41598-025-22336-0 (PMC12583566; doi:10.1038/s41598-025-22336-0)
Supplement: Supplementary file 2 — Supplementary Material 2 [file 41598_2025_22336_MOESM2_ESM.docx]

**Appendix**

## **Post Hoc Comparisons of Emotions**

The table presents the results of a post hoc comparision between different emotions. The Bonferroni correction was applied for multiple comparision. Significant comparisions are indicated with * p < .05, ** p < .01, *** p < .001

## **Table A1**

|  | | | | | | | | | | | | | |
| --- | --- | --- | --- | --- | --- | --- | --- | --- | --- | --- | --- | --- | --- |
| Emotions | |  | | Mean Difference | | SE | | df | | t | p_bonf_ | | |
| Joyful |  | happy |  | -0.032 |  | 0.009 |  | 398 |  | -3.511 |  | 0.022 | * |
|  |  | amusing |  | -0.044 |  | 0.009 |  | 398 |  | -4.682 |  | < .001 | *** |
|  |  | energizing |  | -0.015 |  | 0.009 |  | 398 |  | -1.667 |  | 1.000 |  |
|  |  | dreamy |  | -0.020 |  | 0.009 |  | 398 |  | -2.254 |  | 1.000 |  |
|  |  | relaxing |  | -0.052 |  | 0.010 |  | 398 |  | -5.118 |  | < .001 | *** |
|  |  | neutral |  | -0.118 |  | 0.015 |  | 398 |  | -7.734 |  | < .001 | *** |
|  |  | sad |  | -0.028 |  | 0.010 |  | 398 |  | -2.868 |  | 0.195 |  |
|  |  | annoying |  | -0.021 |  | 0.009 |  | 398 |  | -2.228 |  | 1.000 |  |
|  |  | anxious |  | -0.006 |  | 0.008 |  | 398 |  | -0.706 |  | 1.000 |  |
| Happy |  | amusing |  | -0.012 |  | 0.011 |  | 398 |  | -1.098 |  | 1.000 |  |
|  |  | energizing |  | 0.017 |  | 0.010 |  | 398 |  | 1.787 |  | 1.000 |  |
|  |  | dreamy |  | 0.012 |  | 0.010 |  | 398 |  | 1.270 |  | 1.000 |  |
|  |  | relaxing |  | -0.020 |  | 0.012 |  | 398 |  | -1.702 |  | 1.000 |  |
|  |  | neutral |  | -0.086 |  | 0.016 |  | 398 |  | -5.409 |  | < .001 | *** |
|  |  | sad |  | 0.004 |  | 0.011 |  | 398 |  | 0.329 |  | 1.000 |  |
|  |  | annoying |  | 0.011 |  | 0.010 |  | 398 |  | 1.095 |  | 1.000 |  |
|  |  | anxious |  | 0.026 |  | 0.010 |  | 398 |  | 2.695 |  | 0.331 |  |
| Amusing |  | energizing |  | 0.029 |  | 0.011 |  | 398 |  | 2.687 |  | 0.338 |  |
|  |  | dreamy |  | 0.024 |  | 0.011 |  | 398 |  | 2.200 |  | 1.000 |  |
|  |  | relaxing |  | -0.008 |  | 0.012 |  | 398 |  | -0.628 |  | 1.000 |  |
|  |  | neutral |  | -0.074 |  | 0.017 |  | 398 |  | -4.342 |  | < .001 | *** |
|  |  | sad |  | 0.016 |  | 0.011 |  | 398 |  | 1.354 |  | 1.000 |  |
|  |  | annoying |  | 0.023 |  | 0.011 |  | 398 |  | 2.128 |  | 1.000 |  |
|  |  | anxious |  | 0.038 |  | 0.010 |  | 398 |  | 3.643 |  | 0.014 | * |
| Energizing |  | dreamy |  | -0.005 |  | 0.009 |  | 398 |  | -0.550 |  | 1.000 |  |
|  |  | relaxing |  | -0.037 |  | 0.011 |  | 398 |  | -3.351 |  | 0.040 | * |
|  |  | neutral |  | -0.103 |  | 0.016 |  | 398 |  | -6.405 |  | < .001 | *** |
|  |  | sad |  | -0.014 |  | 0.011 |  | 398 |  | -1.271 |  | 1.000 |  |
|  |  | annoying |  | -0.006 |  | 0.010 |  | 398 |  | -0.620 |  | 1.000 |  |
|  |  | anxious |  | 0.009 |  | 0.010 |  | 398 |  | 0.933 |  | 1.000 |  |
| Dreamy | | relaxing |  | -0.032 |  | 0.011 |  | 398 |  | -2.916 |  | 0.168 |  |
|  |  | neutral |  | -0.098 |  | 0.016 |  | 398 |  | -6.280 |  | < .001 | *** |
|  |  | sad |  | -0.009 |  | 0.011 |  | 398 |  | -0.769 |  | 1.000 |  |
|  |  | annoying |  | -7.749×10^-4^ |  | 0.010 |  | 398 |  | -0.077 |  | 1.000 |  |
|  |  | anxious |  | 0.014 |  | 0.009 |  | 398 |  | 1.538 |  | 1.000 |  |
| Relaxing |  | neutral |  | -0.066 |  | 0.017 |  | 398 |  | -3.850 |  | 0.006 | ** |
|  |  | sad |  | 0.023 |  | 0.012 |  | 398 |  | 1.918 |  | 1.000 |  |
|  |  | annoying |  | 0.031 |  | 0.012 |  | 398 |  | 2.638 |  | 0.390 |  |
|  |  | anxious |  | 0.046 |  | 0.010 |  | 398 |  | 4.524 |  | < .001 | *** |
| Neutral |  | sad |  | 0.089 |  | 0.016 |  | 398 |  | 5.430 |  | < .001 | *** |
|  |  | annoying |  | 0.097 |  | 0.016 |  | 398 |  | 6.070 |  | < .001 | *** |
|  |  | anxious |  | 0.112 |  | 0.016 |  | 398 |  | 7.169 |  | < .001 | *** |
| Sad |  | annoying |  | 0.008 |  | 0.011 |  | 398 |  | 0.707 |  | 1.000 |  |
|  |  | anxious |  | 0.023 |  | 0.011 |  | 398 |  | 2.137 |  | 1.000 |  |
| Annoying | | anxious |  | 0.015 |  | 0.010 |  | 398 |  | 1.543 |  | 1.000 |  |
|  | | | | | | | | | | | | | |
| * p < .05, ** p < .01, *** p < .001 | | | | | | | | | | | | | |
| Note.  P-value adjusted for comparing a family of 45 estimates. | | | | | | | | | | | | | |
